# The Atypical Chemokine Receptor Ackr2 Constrains NK Cell Migratory Activity and Promotes Metastasis

**DOI:** 10.4049/jimmunol.1800131

**Published:** 2018-08-29

**Authors:** Christopher A. H. Hansell, Alasdair R. Fraser, Alan J. Hayes, Marieke Pingen, Claire L. Burt, Kit Ming Lee, Laura Medina-Ruiz, Demi Brownlie, Megan K. L. Macleod, Paul Burgoyne, Gillian J. Wilson, Robert J. B. Nibbs, Gerard J. Graham

**Affiliations:** Chemokine Research Group, Institute of Infection, Immunity, and Inflammation, University of Glasgow, Glasgow G12 8TA, United Kingdom

## Abstract

Chemokines have been shown to be essential players in a range of cancer contexts. In this study, we demonstrate that mice deficient in the atypical chemokine receptor Ackr2 display impaired development of metastasis in vivo in both cell line and spontaneous models. Further analysis reveals that this relates to increased expression of the chemokine receptor CCR2, specifically by KLRG1^+^ NK cells from the Ackr2^−/−^ mice. This leads to increased recruitment of KLRG1^+^ NK cells to CCL2-expressing tumors and enhanced tumor killing. Together, these data indicate that Ackr2 limits the expression of CCR2 on NK cells and restricts their tumoricidal activity. Our data have important implications for our understanding of the roles for chemokines in the metastatic process and highlight Ackr2 and CCR2 as potentially manipulable therapeutic targets in metastasis.

## Introduction

Dissemination of cells from a primary tumor site is essential for the establishment of metastasis, which is responsible for the majority of cancer-related deaths (1). However, our limited understanding of the biology of the metastatic process has severely hampered therapeutic development. Recently, chemokines and their receptors have emerged as important players in the metastatic process (2, 3). The chemokine/receptor axis is pharmacologically manipulable (4) and therefore represents a potential therapeutic target in the context of metastasis.

Chemokines are biochemically related and characterized by the presence of variations on a conserved cysteine motif in their mature sequences. They are named, as CC, CXC, XC, or CX3C, according to the variant of this motif that they possess (5). Chemokines are classified as being either inflammatory or homeostatic according to the immune contexts in which they function (6, 7) and interact with target cells by binding to cognate 7-transmembrane–spanning G-protein–coupled receptors (8). Chemokines and their receptors are essential for regulating the migration of inflammatory and homeostatic leukocytes in a range of physiological and pathological contexts. In metastasis, chemokine receptors such as CXCR4, CCR7, and CCR10 have been implicated in controlling the tissue tropism of metastasizing cells (3). Furthermore, once metastatic cells reach an appropriate tissue, there is clear evidence that they extravasate from the vasculature using a mechanism that relies in part on prometastatic macrophages (9). The monocytic precursors for these macrophages express the chemokine receptor CCR2, and their recruitment to the site of extravasation is dependent on expression of its cognate ligand CCL2. Therefore, chemokines and their receptors are important players in metastasis.

Chemokine function in vivo is also regulated by the atypical chemokine receptors (ACKRs) (10). There are currently four members of this family: Ackr1 (DARC), Ackr2 (D6), Ackr3 (CXCR7), and Ackr4 (CCRL1) (11), which are characterized by an atypical signaling response to chemokine binding and an inability to directly support leukocyte migration. Ackr2 (12) displays promiscuous binding of inflammatory CC chemokines, all of which are ligands for CCRs 1–5. Ackr2 is prominently expressed on lymphatic endothelial cells in resting tissues (13) as well as on some leukocytes (14–16). In addition, within inflamed skin, it is strongly expressed on epidermal cells (17). Ackr2 acts as a scavenger receptor for its ligands, internalizing them and targeting them for intracellular destruction (18, 19). It therefore has an important role in the resolution of chemokine-driven inflammatory responses in the tissues in which it is expressed (10). Ackr2 has also been implicated in the regulation of inflammation-dependent cancer development in skin (20) and colorectal cancer models (21).

Interestingly, one of the key ligands for Ackr2 is CCL2, which, as mentioned above, is strongly implicated in metastasis. We have therefore examined the involvement of Ackr2 in the metastatic process using a range of metastatic models. In this study, we show that Ackr2^−/−^ mice display profoundly impaired metastatic development in both cell line and spontaneous models of metastasis. Further analysis demonstrates that this is a consequence of hyperresponsiveness of KLRG1^+^ NK cells from Ackr2^−/−^ mice to CCL2, which is expressed by the developing metastatic lesions. This leads to increased recruitment of NK cells from Ackr2^−/−^ mice to the developing lesions and enhanced tumor killing. Our data highlight a key interaction between Ackr2 and CCR2 in regulating metastasis and suggest that driving increased CCR2 expression in NK cells or isolation and expansion of CCR2^HI^ NK cells may provide an effective antitumor cell therapeutic product in the context of primary tumors with a high risk of metastatic spread.

## Materials and Methods

### Mice

Animals were cohoused in individual ventilated cages in a barrier facility proactive in environmental enrichment. Ackr2‐deficient mice (22) were bred in‐house (C57BL/6 background); wild type (WT) C57BL6/J mice were from Charles River Research Models and Services. Polyoma middle T (PyMT) transgenic mice (23) (FVB background) were kindly provided by Dr. K. Blyth. Ackr2‐deficient mice (FVB background) were crossed with PyMT mice to yield Ackr2^−/−^ × PyMT mice. All experimental mice were sex matched and used between the ages of 6 and 9 wk. Animal work was carried out with ethical approval from University of Glasgow under the revised Animal (Scientific Procedures) Act 1986 and the European Union Directive 2010/63/EU. All experiments were performed in accordance with relevant guidelines and regulations. The numbers of animals used in each experiment are noted in the relevant figure legends.

### Cell culture

B16F10 and Lewis lung carcinoma cells were obtained from European Collection of Authenticated Cell Cultures and maintained in RPMI 1640 (Life Technologies), 10% FCS, 2 mM l-glutamine (Life Technologies), 100 U/ml penicillin/streptomycin (Sigma-Aldrich). YAC-1 cells were purchased from the American Type Culture Collection and cultured in suspension as above. All cell lines were cultured under sterile conditions at 37°C in 5% CO_2_ and enzymatically dispersed and washed before administration.

### NK cell isolation

NK cells were pre-enriched from lung or spleen (as indicated) using the NK Cell Isolation Kit II, mouse (Miltenyi Biotec) as per the manufacturer’s instructions. TCRβ^−ve^ NK1.1^+^ CD11b^+^ KLRG1^+^ NK cells were then purified to ≥95% purity using a FACS sorter (BD FACSAria I or III; Becton Dickinson).

### Adoptive transfers

In the therapeutic NK transfer experiment at day 0, recipient WT C57BL/6J mice received 5 × 10^5^ B16F10 cells i.v. One day posttumor administration, mice received 2 × 10^5^ WT or Ackr2^−/−^ TCRβ^−ve^ NK1.1^+^ CD11b^+^ KLRG1^+^ NK cells i.v. Mice were culled at day 14, and pulmonary metastatic deposits were enumerated. In competitive transfer experiments, B16F10 melanoma cells were labeled with 10 μM CMTPX (Life Technologies) per the manufacturer’s instructions. A total of 1 × 10^6^ labeled B16F10 cells were injected i.v. on day 0. On day 2, TCRβ^−ve^ NK1.1^+^ CD11b^+^ KLRG1^+^ NK cells were FACS purified from either WT or Ackr2^−/−^ mice and differentially labeled with CFSE (Life Technologies) or 5 μM Cell Proliferation Dye eFluor 670 (eBioscience). The mice were injected i.v. with at least 1.3 × 10^6^ mixture of labeled WT and Ackr2^−/−^ NK cells. Mice were culled 24 h later, and tissues were taken. Lungs were perfused as described above and inflated using 1% low–melting point agar. Lungs and spleen were then placed in 1% paraformaldehyde for 24 h and transferred to 30% sucrose for a further 24 h before freezing in Cryomatrix (Thermo Fisher Scientific). Frozen sections (8 μm) were cut on a Shandon Cryotome FSE (Thermo Fisher Scientific), and fluorescent images were acquired across nine separate tissue sections using a Zeiss Axio Imager M2 epifluorescence microscope with AxioVision software (release 4.8.2 06‐2010/Zeiss ZEN 2012 Blue edition). The NK ratio observed in the spleen was used to calculate the “input” ratio. Images taken at random or centered on tumor cells were used to calculate “random field” versus “tumor” ratios.

### Chemotaxis assay

TCRβ^−ve^ NK1.1^+^ CD11b^+^ KLRG1^+^ NK cells were resuspended in chemotaxis buffer (RPMI 1640 and 0.5% BSA). Approximately 5 × 10^5^ cells were placed in the upper chambers of 24-well transwell plates (3-μm filters; Corning) above 600 μl of chemotaxis buffer with or without chemokine (PeproTech) and incubated at 37°C and 5% CO_2_ for 3 h. Migrated cells were enumerated by FACS using CountBrite beads (Invitrogen) and converted into the percentage of input for each subset.

### NK cell-killing assays

KLRG1^+^ NK cells were FACS purified from Ackr2^−/−^ and WT spleens using a BD ARIA III flow sorter to a purity of ∼99%. These were seeded at a density of 5 × 10^4^ NK cells per well of a 96-well round-bottomed plate in RPMI 1640 (Life Technologies), 10% FCS, 2 mM l-glutamine (Life Technologies), and 100 U/ml penicillin/streptomycin (Sigma-Aldrich). YAC-1 cells were labeled with CFSE (Life Technologies) and cocultured with NK cells at the indicated ratios in the presence or absence of Protein Transport Inhibitor Cocktail (Affymetryx eBioscience) per the manufacturer’s instructions. After 5 h incubation, cocultures were stained using specific Abs for intracellular IFN-γ (clone XMG1.2; Affymetryx eBioscience) and the exocytosis marker CD107 (clone 1D4B; BioLegend). NK cell production of IFN-γ and NK cell–induced YAC-1 cell death was subsequently determined by flow cytometry. Killing of B16F10 cells by NK cells in vitro was assessed using the same methodology as for YAC-1 cells.

### Histology and immunohistochemistry

Lungs and other tissues were fixed in 4% paraformaldehyde overnight before transfer to 70% ethanol. Tissues were embedded in paraffin wax using the Shandon Citadel 1000 (Thermo Shandon). Sections (4-μm thick) were cut using a Shandon Finesse 325 Microtome. Resultant sections were stained with Giemsa, H&E, or toluidine blue for subsequent analysis. For the PyMT study, sections from eight representative regions through each of the lungs were chosen, and all tumors were enumerated in the tissue. Giemsa stain was sufficient to discriminate the metastatic colonies from normal lung tissue. For immunohistochemistry, slides were deparaffinized, rehydrated through alcohols, and stained using specific Abs. To stain for CCL2, slides were boiled for 30 min in Tris-EDTA after blocking with 20% goat serum (Vector Laboratories). Goat polyclonal anti-mouse CCL2 (M-18; Santa Cruz Biotechnology) was used as the primary Ab, followed by an anti-goat HRP (Vector Laboratories) secondary Ab. In the case of Mac-2, Ag retrieval was not required. Slides were blocked using 20% goat serum (Vector Laboratories), followed by rat anti-mouse Mac-2 Ab (clone M3/38) (CEDARLANE), goat anti-rat IgG biotin (Vector Laboratories), and finally ExtrAvidin-Peroxidase labeled streptavidin-biotin reagent (Sigma-Aldrich).

### Flow cytometric analysis

Spleen cells were harvested, and single-cell suspensions were prepared by mechanical disruption through a 40-μM EASYstrainer (Greiner Bio-One). RBCs were subsequently lysed by resuspending the cell pellet in 1 ml RBC lysis buffer (eBioscience) for 1 min before washing in FACS buffer (PBS, 1% FCS, 0.02% sodium azide, 5 mM EDTA). A total of 1–3 × 10^6^ cells per well of a 96-well round-bottomed plate were incubated with 50 μl of a 5 μg/ml solution of Fc block (BioLegend) for 15 min on ice, washed twice with FACS buffer, and stained with fluorescently labeled Abs (various concentrations) and Via-Probe (BD Biosciences) or fixable viability dye eFluor 506 or 780 (eBioscience) (to exclude dead cells). Bone marrow samples were flushed from the bone using a syringe with a 26-gauge needle charged with FACS buffer. Single-cell suspensions were prepared, and RBC were lysed as per the spleen. For analysis of blood samples, RBC were lysed using ammonium chloride solution (STEMCELL Technologies), according to the manufacturer’s instructions, immediately prior to staining. Lungs were perfused with PBS/2 mM EDTA, and the tissue was removed and finely minced with scissors before resuspending in digestion mixture (3.2 mg/ml Dispase [Roche], 0.4 mg/ml Collagenase P [Roche], and 0.2 mg/ml DNase I [Life Technologies]) and incubating at 37°C for 40 min before passing through a 40-μM EASYstrainer (Greiner Bio-One). RBCs were lysed as previously described for the spleen. For intracellular cytokine staining, isolated cells were incubated for 5 h in RPMI 1640 10% FCS, 2 mM l-glutamine, 100 U/ml penicillin/streptomycin at 37°C, 5% CO_2_ in the presence of 1× Cell Stimulation Cocktail (plus protein transport inhibitors; eBioscience). Cultured cells were subsequently stained for surface Ags, as above, and dead cells were excluded using viability dye eFluor 506 or 780 (eBioscience). Cells were fixed using IC Fixation Buffer (eBioscience), permeabilized using Permeabilization Buffer (eBioscience), and stained for intracellular cytokines. Positive populations were defined on the basis of size (to exclude doublet populations), viability (i.e., viability dye negative), and Fluorescence Minus One isotype controls. Data were analyzed using FlowJo software (Tree Star).

### Abs used for flow cytometry

Abs against the following surface markers were used, with clone names in parentheses: CD107a (ID4B), TCRβ (H57597), KLRG1 (2F1/KLRG1), NK1.1 (PK136), NKp46 (29A1.4), CD11b (M1/70), CD49f (GoH3), Ly-6C (HK1.4), and CD11c (N418), all BioLegend Abs; CD45 (30F11), CD49b (DX5), Granzyme B (NGZB), and F4/80 (BM8), all eBioscience Abs; SiglecF (E50-2440; BD Biosciences) with a variety of conjugated fluorochromes; and Streptavidin Qdot605 (Life Technologies); appropriate isotype controls were purchased from BD Biosciences or eBioscience.

### Tumor models

#### B16F10 metastasis model.

Typically, 5 × 10^5^ B16F10 cells (kindly provided by Dr. D. Greenhalgh, University of Glasgow) were injected i.v., and mice were culled and organs were harvested at various time points up to a maximum of 14 d postinoculation. In experiments allowed to proceed to day 14, blinded counts of visible melanic surface tumors were taken. In experiments in which tumor cells were fluorescently labeled, 1 × 10^6^ cells were used to improve detectability at early time points. CCR2 blockade experiments were performed using the small molecule inhibitor CCX872-22A versus a hydroxylpropyl methylcellulose carrier control, both kindly provided by ChemoCentryx. Mice received a daily dose of 5 mg/kg s.c. for the duration of the model starting 2 d prior to injection of B16F10 cells. To deplete monocyte/macrophage lineages, mice were treated with clodronate liposomes versus a PBS liposome control (Liposoma; https://clodronateliposomes.com/?v=7516fd43adaa). Mice were treated i.v. every 4 d with 75 μl of a 5 mg/ml solution of liposomes, starting 2 d prior to the administration of B16F10 cells, receiving their final dose at day 10 post-B16F10 cell administration before cull at day 14. To deplete NK cells, mice received 200 μl i.p. of a 1 mg/ml solution of NK1.1 Ab (clone PK136) versus IgG2a isotype control (both eBioscience). Mice were injected with the Ab 2 d prior to B16F10 treatment and then at days 4 and 10 prior to cull at day 14.

#### PyMT spontaneous metastasis model.

In this model, female mice spontaneously develop mammary epithelial tumors by ∼90 d of age, and mice were monitored throughout to assess tumor growth in the mammary tissue using tissue calipers. Once any single tumor within one of the eight mammary pads reached more than 10 mm in any axis, the mouse was culled, and total mammary tumor burden was calculated. Survival, as reported in Fig. 2, is defined as time to cull. During the period of primary tumor growth, there is also metastatic colonization of the lungs, and lung tissue was collected for histological assessment.

### Lentiviral production and transduction of J774.2 cells

Lentiviral particles were produced as described previously (24) with some modifications. In brief, 4 T-150 flasks of HEK293T cells were transfected with 50 μg of lentiviral vector (containing ACKR2) and the helper plasmids PMD2.G and pΔ8.91(17.5 and 32.5 μg, respectively). Supernatant containing the lentiviral particles was collected after 48 and 72 h of incubation. Lentiviral particles were concentrated using PEG 8000 (Promega) at a final concentration 70 g/l. Viral titers were determined by quantitative PCR (qPCR), using primers 5′ long terminal repeat forward (5′-TGTGTGCCCGTCTGTTGTGT-3′) and 5′ long terminal repeat reverse (5′-GAGTCCTGCGTCGAGAGAGC-3′).

For the transduction of J774.2 cells, 6 × 10^4^ cells were plated onto each chamber of a four-chamber slide and cultured overnight in DMEM. Twenty-four hours later, medium was replaced with 0.5 ml of DMEM containing the lentiviral particles (multiplicity of infection 50) and polybrene (8 μg/ml). Twenty-four hours posttransduction, lentiviral particles were removed and replaced with fresh DMEM. The expression of ACKR2 and CCR2 was analyzed 48 h posttransduction.

### qPCR

RNA was extracted using RNeasy columns with DNase treatment (Qiagen), and the amount of RNA was quantified on a Nanodrop 1000 Spectrophotometer (Thermo Fisher Scientific). cDNA was synthesized using AffinityScript Multiple Temperature cDNA Synthesis Kit (Agilent Technologies). For all qPCRs, a final concentration of 0.2-μM primers was used for each PCR set up using PerfeCTa SYBR Green FastMix and ROX qPCR Master Mix (Quanta BioSciences). qPCRs were performed on a Prism 7900HT Fast Real-Time PCR System (Applied Biosystems) or a Real-Time PCR system (Applied Biosystems). The thermal cycles for qPCR of GAPDH and CCR2 were 95°C (3 min) for one cycle and 95°C (3 s) and 60°C (30 s) for 40 cycles. Relative expression was calculated using serial dilutions of cDNA standards. Primer sequences designed for qPCR and for producing cDNA standards were designed using Primer3 software (http://frodo.wi.mit.edu/cgi-bin/primer3/primer3_www.cgi). The following primers were used: mouse ACKR2, 5′-TTCTCCCACTGCTGCTTCAC-3′ and 5′-TGCCATCTCAACATCACAGA-3′; mouse CCR2 QPCR, 5′-TGTGGGACAGAGGAAGTGG-3′ and 5′-GGAGGCAGAAAATAGCAGCA-3′; and mouse CCR2 standards, 5′-AGGGGAGAGCAGAAGGCTAA-3′ and 5′-CCCAGGAAGAGGTTGAGAGA-3′.

### Chemokine uptake assay

Chemokine receptor expression was detected in leukocytes using fluorescently labeled chemokines as described previously (14, 16, 25, 26) and as shown diagrammatically in Supplemental Fig. 2. Leukocytes (≤2 × 10^6^ per well) were isolated as described above and cultured (37°C, 60 min) in 50 μl complete medium (+20 mM HEPES [pH 7.4]) containing 25 nM Alexa Fluor 647–coupled human CCL2 or CCL22 to detect CCR2 or Ackr2, respectively (Alexa-CCL2 or Alexa-CCL22 [Almac]). Cells cultured in this way internalize Alexa-chemokine via chemokine receptors or nonspecific mechanisms and become fluorescent. Specific chemokine receptor function is identified by comparing WT populations with those derived from receptor knockouts or comparison with cells cultured in the presence of ± 250 nM unlabeled mouse chemokine competitor (typically CCL12; PeproTech). Cells were then briefly washed before immunostaining. To detect ACKR2 activity in lung stroma, Alexa-CCL22 was administered either intratracheally or i.v. Intratracheal detection was performed on euthanized mice. The trachea was exposed, and the lungs were inflated with 1 ml of 25-nM Alexa-CCL22 ± 250 nM unlabeled mouse chemokine competitor (PeproTech). The trachea was tied off with surgical thread, and the intact inflated lungs and trachea were removed and incubated in a bath of RPMI 1640 (37°C, 60 min). Following this period, cells were isolated enzymatically as described above and stained for flow cytometric analysis. i.v. detection required the injection of 1 μg Alexa-CCL22 i.v. Mice were euthanized 2 h postinjection, and tissues were harvested as described above and stained for flow cytometric analysis.

### Statistical analysis

The statistical tests used are indicated in the relevant figure legends. In all instances, statistical tests were carried out using GraphPad Prism software. All quantitative data are presented as mean ± SD.

## Results

### Ackr2^−/−^ mice display impaired pulmonary metastasis

**FIGURE 1.**
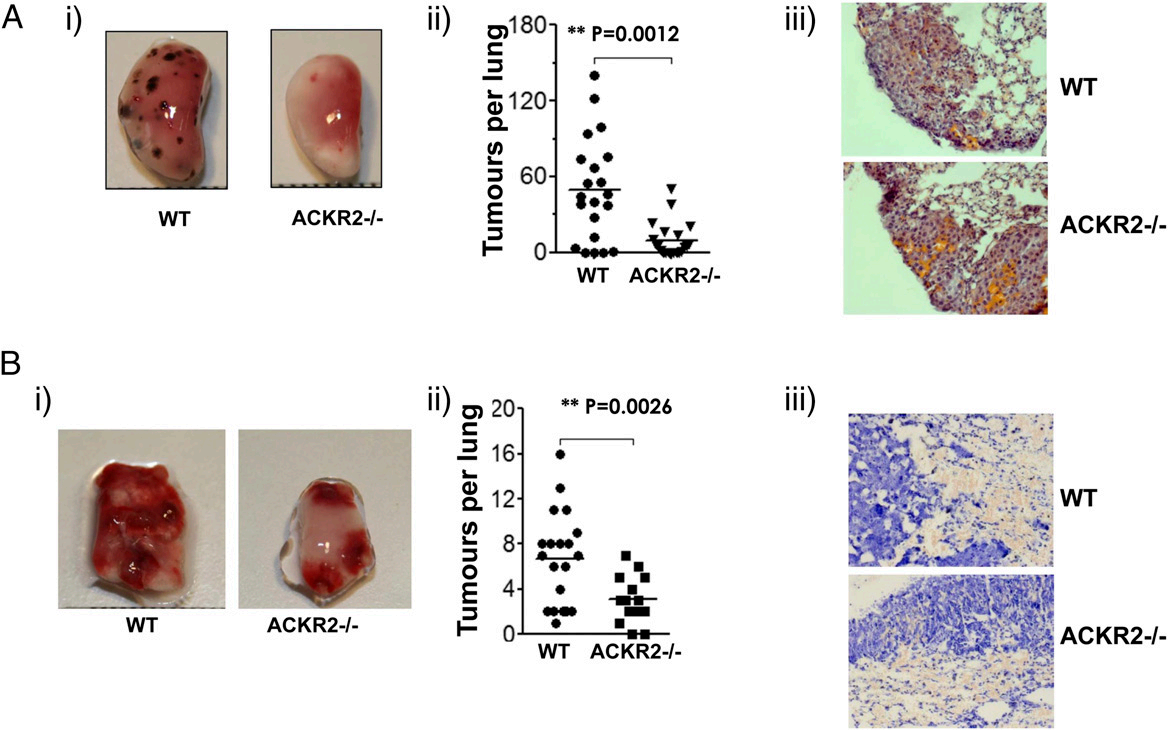
Impaired metastasis development in Ackr2^−/−^ mice. (**A**) Ackr2^−/−^ mice display impaired B16F10 melanoma cell metastasis to the lungs. This is apparent on gross examination of the lungs (A**i**) and enumeration of metastatic deposits (*n* = 21) (A**ii**). Histological analysis revealed no differences in overall tumor architecture in WT and Ackr2^−/−^ lungs (A**iii**). (**B**) Ackr2^−/−^ mice display impaired Lewis lung carcinoma cell metastasis to the lungs. This is apparent on gross examination (B**i**) and enumeration of metastatic deposits (B**ii**). Histological analysis revealed no differences in overall tumor architecture in WT and Ackr2^−/−^ lungs (*n* ≥ 15) (B**iii**). H&E stained, original magnification ×5. Unpaired *t* test was used to assess significance in all cases. Each experiment was carried out at least three times with similar results.

To examine roles for Ackr2 in metastasis, we initially used the B16F10 melanoma cell line model. These cells are injected i.v. and give rise to easily identifiable, melanic, pulmonary metastatic foci. As shown (Fig. 1Ai), although WT mice displayed numerous pulmonary metastatic deposits, Ackr2^−/−^ mice displayed markedly fewer metastases. Quantification revealed a highly significant reduction in metastatic deposits in the lungs of Ackr2^−/−^ compared with WT mice (Fig. 1Aii), and this is further supported by images of whole lungs from B16F10-treated WT (Supplemental Fig. 1Ai) and Ackr2^−/−^ (Supplemental Fig. 1Aii) mice. These images demonstrate numerous metastatic deposits throughout the WT lung section compared with the very small numbers of deposits identified in the Ackr2^−/−^ lungs section. Notably, where metastases did arise in Ackr2^−/−^ mice, they were histologically indistinguishable from those in WT mice (Fig. 1Aiii).

We next examined metastatic development in response to i.v. injection of Lewis lung carcinoma cells (27). Again, Ackr2^−/−^ mice displayed impaired pulmonary metastasis development (Fig. 1Bi) with significantly fewer metastatic deposits compared with WT mice (Fig. 1Bii). Low-magnification images of lungs from Lewis lung carcinoma–bearing WT (Supplemental Fig. 1Bi) and Ackr2^−/−^ (Supplemental Fig. 1Bii) mice revealed extensive disruption of the normal lung architecture by large tumor deposits in WT lungs but only small isolated tumor deposits, among otherwise normal lung tissue, in Ackr2^−/−^ lungs. When they developed, the metastases in Ackr2^−/−^ lungs were histologically indistinguishable from those in WT lungs (Fig. 1Biii). Thus, Ackr2^−/−^ mice display impaired pulmonary metastatic development in two separate cell line models of metastasis.

### Ackr2^−/−^ mice display impaired development of spontaneous metastasis

**FIGURE 2.**
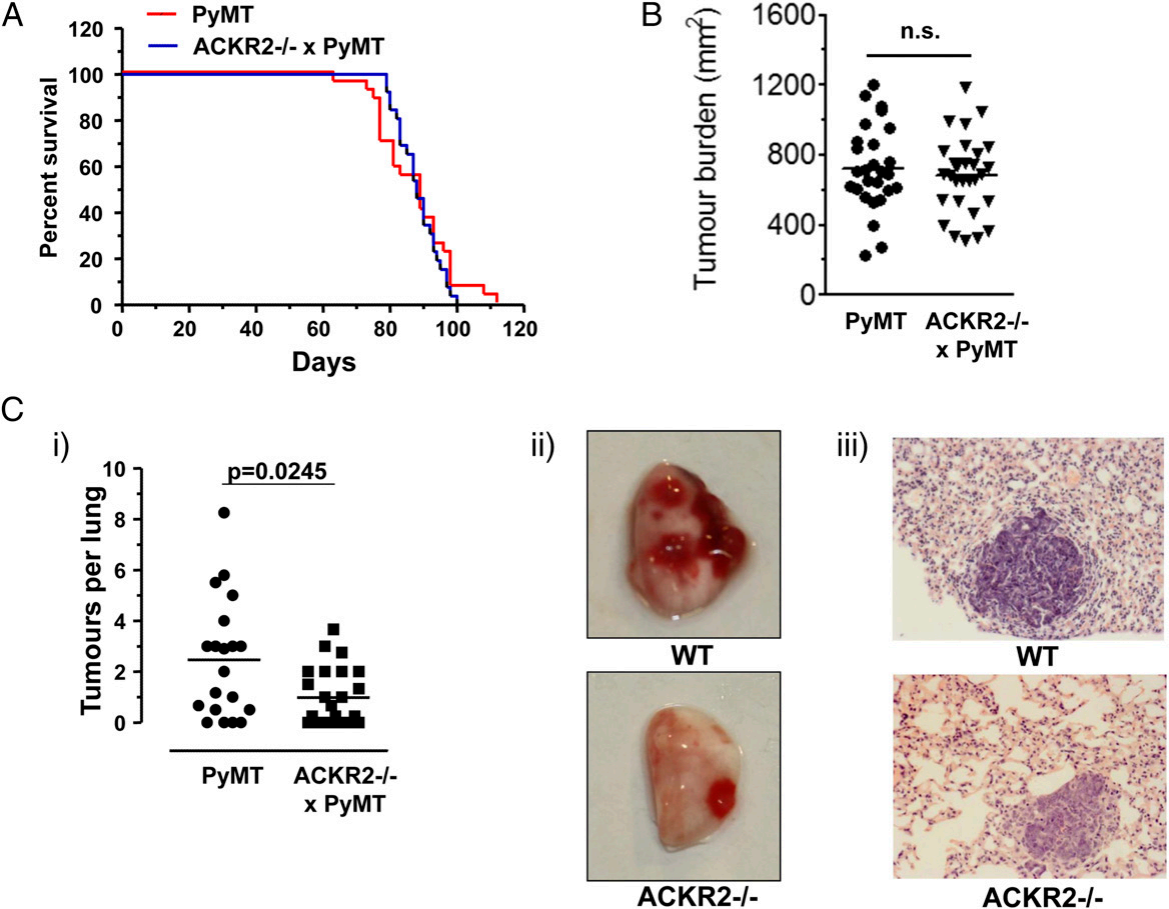
Impaired development of spontaneous metastases in Ackr2^−/−^ mice. WT PyMT and Ackr2^−/−^ × PyMT mice display no differences in percentage survival measured as time to cull (*n* ≥ 26) (**A**) or size of primary tumor burden in the mammary glands (*n* ≥ 27) (**B**). However, Ackr2^−/−^ × PyMT mice develop fewer metastatic deposits. This is apparent on gross examination (**Cii**) and enumeration of numbers of metastatic deposits (*n* ≥ 20) (**i**). Histological analysis revealed no differences in overall tumor architecture in WT and Ackr2^−/−^ lungs (**iii**). H&E stained, original magnification ×5. Unpaired *t* test was used to assess significance in all cases. This study was carried out twice with similar results.

To examine roles for Ackr2 in metastasis from a primary tumor, we crossed Ackr2^−/−^ mice onto the PyMT background (23). These mice develop a primary mammary carcinoma that metastasizes spontaneously to the lung. In this model, the size of the primary tumor defines time to cull. As shown (Fig. 2A), there was no difference in time to cull of WT or Ackr2^−/−^ mice on the PyMT background, and there were no significant differences in size of the primary tumor burden (Fig. 2B). Importantly, there was a marked and significant reduction in the number of pulmonary metastatic deposits in Ackr2^−/−^ compared with WT mice (Fig. 2Ci). Gross images of whole lungs (Fig. 2Cii) revealed extensive large metastatic deposits in WT lungs compared with very small numbers of metastatic deposits in Ackr2^−/−^ lungs. Where metastatic deposits did develop in Ackr2^−/−^ × PyMT mice, they were histologically indistinguishable from those developing in WT/PyMT mice (Fig. 2Ciii). Thus, Ackr2 also contributes to metastasis development in a model of spontaneous pulmonary metastasis.

### Ackr2 does not regulate initial tumor seeding into the lung

**FIGURE 3.**
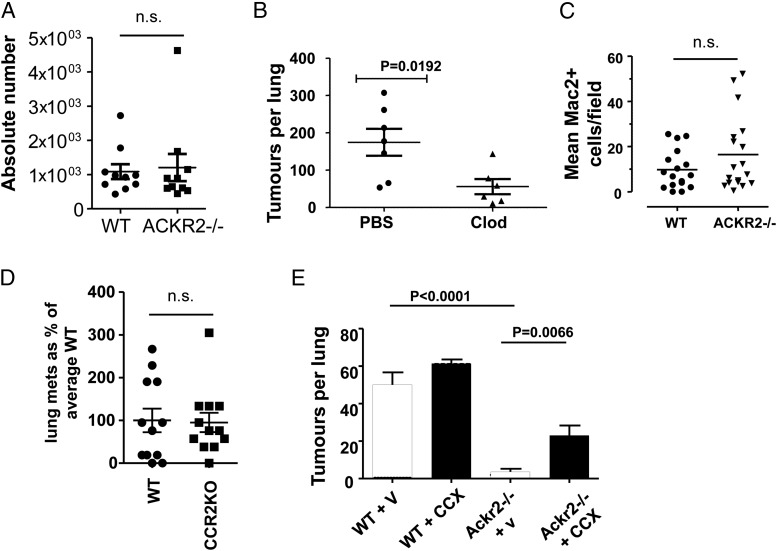
Metastatic cell seeding to the lung is independent of Ackr2. (**A**) CellTracker Orange–labeled B16F10 cells were injected in equal numbers into WT or Ackr2^−/−^ mice. Seventy-two hours later, metastatic cells in lungs were enumerated using flow cytometry. Data represent numbers of recovered dye-labeled cells per lung (*n* = 10). (**B**) Mice received either PBS-containing liposomes or clodronate liposomes to deplete macrophages. B16F10 cells were then administered, and metastatic development was assessed by counting pulmonary metastatic deposits (*n* ≥ 6). (**C**) Macrophage numbers in resting WT and Ackr2^−/−^ lungs were assessed using Mac-2 staining of histological sections. Data are presented as mean Mac-2^+^ cell numbers per visual field (*n* ≥ 17). Unpaired *t* test was used to assess significance. (**D**) WT and CCR2^−/−^ mice were injected with B16F10 cells, and pulmonary metastases were assessed at day 14. Data are presented as percentage of the average WT metastasis score. (**E**) WT or Ackr2^−/−^ mice were administered vehicle (v) or CCR2 blocker (CCX), after which they received B16F10 cells. CCX (or v) was then administered every second day, and lung metastatic deposits developing were enumerated 14 d after B16F10 injection (*n* ≥ 7). All experiments were performed at least twice with similar results. Unpaired *t* test was used to assess significance.

As Ackr2 dampens inflammatory chemokine function, we reasoned that the phenotype in Ackr2^−/−^ mice might relate to altered B16F10 cell migration to inflammatory CC chemokines. However, qPCR analysis of B16F10 cells failed to detect expression of any of the receptors that share ligands with Ackr2 [i.e., CCR1, CCR2, CCR3, CCR4, and CCR5 (10)]. It is therefore unlikely that altered migration of B16F10 cells to inflammatory CC chemokines explains the observed phenotype. Indeed, analysis of the numbers of dye-labeled B16F10 cells in the lungs 72 h after i.v. administration revealed no significant differences between WT and Ackr2^−/−^ mice (Fig. 3A), further suggesting that pulmonary migration/seeding of B16F10 cells is not impaired in Ackr2^−/−^ mice.

It remains possible that although similar numbers of B16F10 cells are detectable in WT and Ackr2^−/−^ lungs, there may be fewer cells entering the lung parenchyma in Ackr2^−/−^ mice. Monocyte/macrophage recruitment has been implicated in metastatic cell extravasation into the lung parenchyma (9), and clodronate liposome administration confirmed their importance for tumor formation in the B16F10 model (Fig. 3B). As Ackr2 scavenges the ligands for the major monocyte/macrophage chemokine receptor CCR2, it may interfere with metastasis development by impairing monocyte/macrophage recruitment through alteration of local chemokine gradients. However, no significant differences in macrophage numbers were detectable between WT and Ackr2^−/−^ lungs (Fig. 3C), suggesting that defective macrophage recruitment is unlikely to explain impaired metastatic development in Ackr2^−/−^ mice. Thus, Ackr2 appears not to regulate initial homing of metastatic cells to the lung.

Given the known involvement of CCL2 [a ligand for CCR2 and Ackr2 (10)] in metastasis, we compared metastasis development in WT and CCR2^−/−^ mice. In contrast to other models (9), CCR2 deficiency had no significant impact on pulmonary metastasis in the B16F10 model (Fig. 3D). This suggests that although macrophages are important for metastasis in this model, they appear not to be fully dependent on CCR2. We also examined effects of a well-characterized pharmacological blocker of CCR2 (28, 29) on metastasis development. Again, CCR2 inhibition had no significant effect on metastasis development in WT mice, confirming the lack of requirement for CCR2 in this model. However, CCR2 inhibition significantly reversed the block in metastasis development in Ackr2^−/−^ mice (Fig. 3E).

Together, these data suggest that although development of metastasis in WT mice is CCR2 independent, a CCR2-dependent population of cells limits metastatic development in Ackr2^−/−^ mice.

### Ackr2^−/−^ KLRG1^+^ NK cells display increased CCR2 expression

**FIGURE 4.**
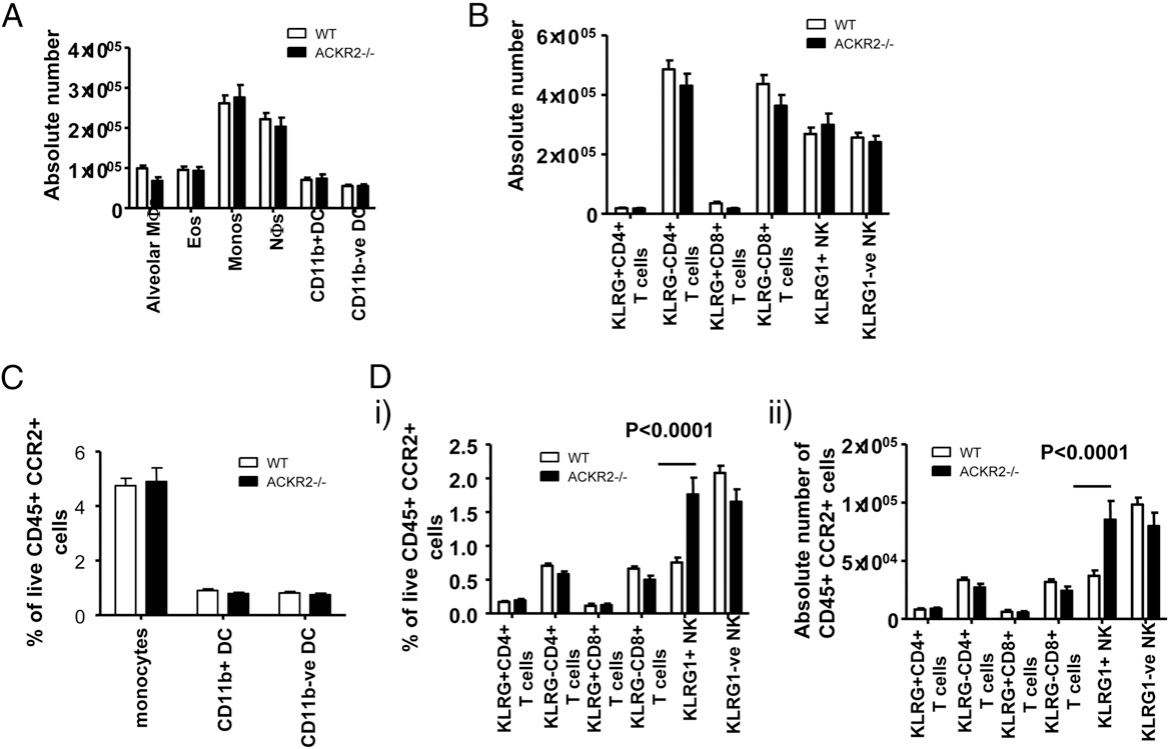
Ackr2^−/−^ mice display differences in CCR2 expression by NK cells. (**A**) Numbers of the indicated myelomonocytic cells in WT and Ackr2^−/−^ lungs 72 h after administration of B16F10 cells. (**B**) Numbers of the indicated lymphoid cell types in WT and Ackr2^−/−^ lungs 72 h after administration of B16F10 cells (*n* = 17). (**C**) Relative percentage of live CD45^+^ myelomonocytic cell subsets that are positive for CCR2 activity as measured by Alexa-CCL2 binding. Data are shown for WT and Ackr2^−/−^ lungs 72 h after B16F10 cell administration (*n* = 17). (**D**) Relative percentage (**i**) and absolute numbers (**ii**) of live CD45^+^ lymphocyte subsets that are positive for CCR2 activity as measured above. Data are shown for WT and Ackr2^−/−^ lungs 72 h after B16F10 cell administration (*n* = 17). All experiments were performed at least twice with similar results. Statistical test used in (B) and (C) was one-way ANOVA with Bonferonni posttest. DC, dendritic cell; Eos, eosinophil; Monos, monocytes; Nɸ, neutrophils.

The above data suggest preferential recruitment of CCR2^+^ tumoricidal cells to Ackr2^−/−^ lungs, implying CCR2^+^ leukocyte involvement. Therefore, we examined levels of inflammatory leukocytes in WT and Ackr2^−/−^ lungs early in the metastatic process (72 h), at which time point we assumed antimetastatic mechanisms to be active. In agreement with the data in Fig. 3, no significant differences were seen in monocyte recruitment, and total numbers of other myelomonocytic cells were also similar (Fig. 4A). In addition, no differences were detected in overall T cell or NK cell numbers (Fig. 4B). To determine whether there were specific differences in CCR2-expressing populations, we selectively measured CCR2^+^ cells within key leukocyte populations using Alexa-CCL2 as described (14, 16, 25, 26) and as detailed in the *Materials and Methods* section and in Supplemental Fig. 2. Notably, although we detected no differences in CCR2^+^ monocytic or dendritic cell numbers between WT and Ackr2^−/−^ lungs (Fig. 4C), we detected a highly significant increase in the apparent number of CCR2^+^ KLRG1^+^ NK cells (but not CCR2^+^ KLRG1^−^ NK cells) in Ackr2^−/−^ lungs (Fig. 4D). Examination of the Alexa-CCL2 mean fluorescence intensity (MFI) for these cells showed that, on a cell-per-cell basis, KLRG1^+^ NK cells from Ackr2^−/−^ mice display higher levels of CCR2 than equivalent WT cells (Supplemental Fig. 3A), indicating that the data in Fig. 4Di reflect increased CCR2 expression on KLRG1^+^ NK cells from Ackr2^−/−^ mice and not increased cell numbers. In agreement with the data in Fig. 4C, flow cytometric analysis failed to detect any differences in CCR2 activity between monocytes from WT and Ackr2^−/−^ mice, confirming selectivity of this phenotype for KLRG1^+^ NK cells (Supplemental Fig. 3B).

The above data were collected from lungs 72 h after i.v. administration of B16F10 cells. However, we see a similar increase in CCR2 expression in Ackr2^−/−^ KLRG1^+^ NK cells in lungs from mice at day 14 [i.e., at the termination of the metastasis model (Supplemental Fig. 3Ci)]. We also note, at this time point, increased numbers of KLRG1^+^ NK cells in the Ackr2 compared with WT lungs (Supplemental Fig. 3Cii), perhaps suggesting that the process of tumor killing by NK cells early in the metastatic process produces an inflammatory environment that leads to secondary recruitment of CCR2^+^ NK cells in Ackr2^−/−^ lungs.

Thus, impaired metastasis development in Ackr2^−/−^ mice involves CCR2-expressing cells, and these mice display increased CCR2 expression on KLRG1^+^ NK cells in metastatic lungs.

### NK cells in resting Ackr2^−/−^ mice display increased CCR2 activity

**FIGURE 5.**
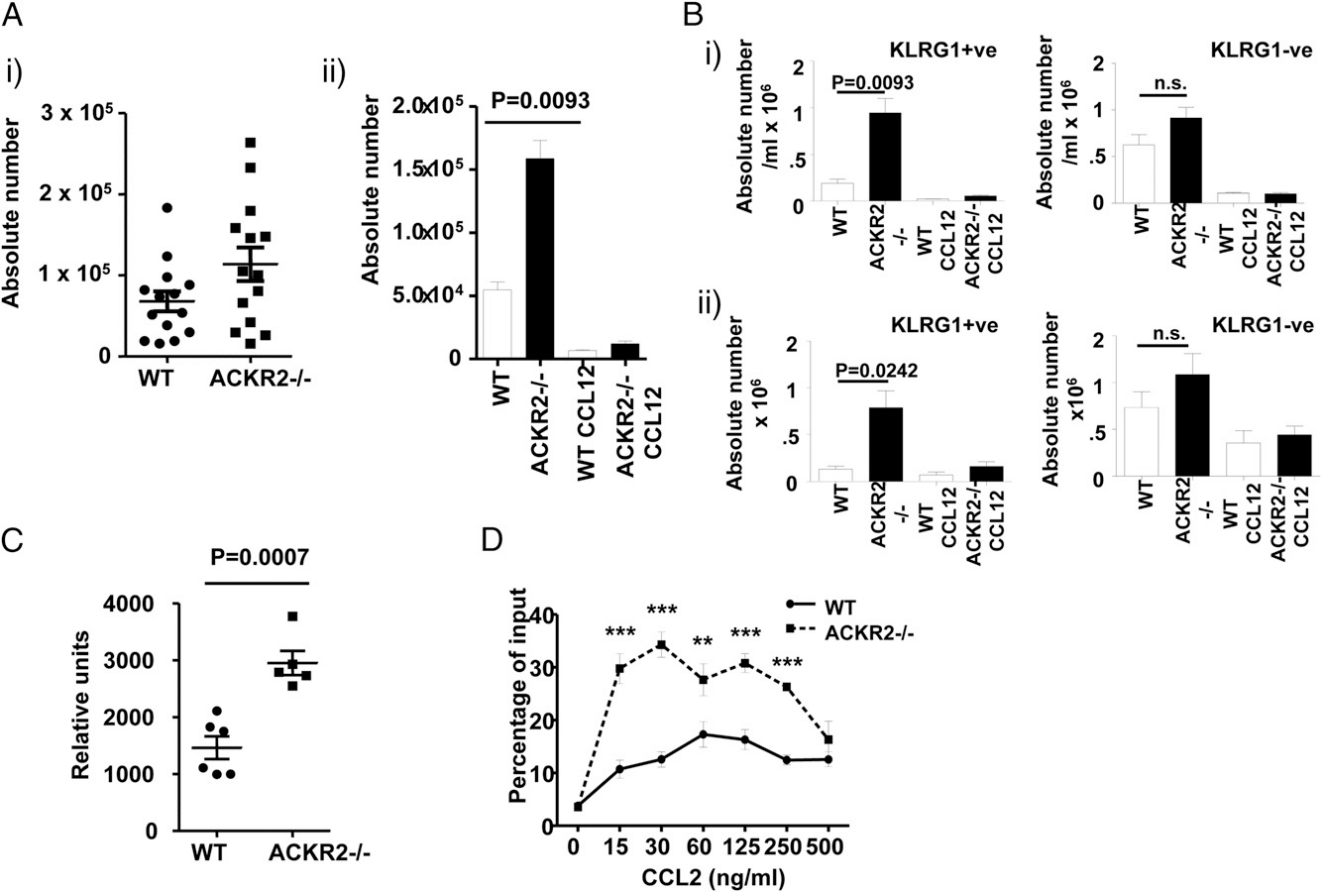
Ackr2^−/−^ KLRG1^+^ NK cells display increased CCR2 activity. (**Ai**) KLRG1^+^ NK cell numbers do not differ between WT and Ackr2^−/−^ lungs at rest (*n* = 14). Unpaired *t* test. (A**ii**) Alexa-CCL2 binding demonstrates increased CCR2 on resting pulmonary Ackr2^−/−^ KLRG1^+^ NK cells compared with WT. Ackr2 specificity of binding was confirmed by competition with excess CCL12 (*n* = 3). (**B**) KLRG1^+^ but not KLRG1^−^ NK cells from blood (B**i**) and spleen (B**ii**) display increased CCR2-specific binding of Alexa-CCL2. This experiment was performed three times with similar results. (**C**) Ackr2^−/−^ KLRG1^+^ NK cells express higher CCR2 transcript levels than equivalent WT cells (*n* ≥ 5). Unpaired *t* test. Data are presented as relative units of expression.(**D**) Ackr2^−/−^ KLRG1^+^ NK cells are more responsive to CCL2 in transwell migration assays than WT counterparts. Migration is measured as percentage of input population migrating toward the indicated CCL2 concentrations (*n* ≥ 3). All experiments were performed at least twice with similar results. *p < 0.05, **p < 0.01, ***p < 0.001.

Next, we compared NK cell populations in resting WT and Ackr2^−/−^ lungs. Again, although no significant differences were detected in total KLRG1^+^ NK cell numbers (Fig. 5Ai), we detected significantly increased CCR2 expression on KLRG1^+^ NK cells in resting Ackr2^−/−^ compared with WT lungs, suggesting a basal difference in NK cell biology in Ackr2^−/−^ mice (Fig. 5Aii). Similar significant differences were also detected in KLRG1^+^ (but not KLRG1^−^) NK cells from Ackr2^−/−^ mice in peripheral blood and spleen (Fig. 5B), suggesting that increased CCR2 activity is common to KLRG1^+^ NK cells in Ackr2^−/−^ mice. Increased CCR2 in KLRG1^+^ NK cells from Ackr2^−/−^ mice was reflected in significantly higher transcript levels (Fig. 5C). In addition, in transwell migration assays, KLRG1^+^ NK cells from Ackr2^−/−^ mice displayed significantly enhanced sensitivity to CCL2 and increased overall levels of migration compared with WT cells (Fig. 5D). Thus, Ackr2^−/−^ mice display systemic and selective upregulation of CCR2 on KLRG1^+^ NK cells.

### ACKR2 does not regulate CCR2 activity on a cell-autonomous basis

**FIGURE 6.**
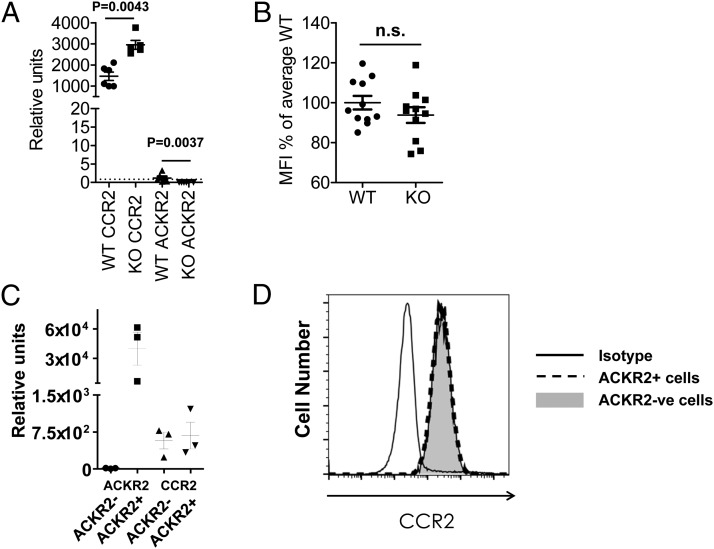
ACKR2 does not directly regulate CCR2 expression. ACKR2 has no cell-autonomous impact on CCR2 expression levels or binding of CCL2. (**A**) qPCR analysis of CCR2 and Ackr2 expression in NK cells from WT and Ackr2^−/−^ mice. Data are expressed as relative units normalized to the housekeeping GAPDH. Data were analyzed using Student *t* test. (**B**) Graph representing Ackr2 expression in KLRG1^+^ splenic NK cells expressed as a percentage of the average WT MFI. Data represent MFI from flow cytometric assessment of Alexa-CCL22 internalization. (*n* = 9 combined from three independent experiments), *t* test. (**C**) Graph displaying relative expression of indicated genes (ACKR2 and CCR2) as determined by qPCR in the J774.2 cell line before (ACKR2^−^) and after (ACKR2^+^) ACKR2 lentiviral transduction (*n* = 4 combined from two individual experiments). (**D**) Representative flow cytometric histogram of CCR2 surface staining of J774.2 before (gray) and after (dashed line) ACKR2 lentiviral transduction relative to isotype control (solid line). Data are presented as mean ± SEM.

We initially hypothesized that Ackr2 had a cell-autonomous effect on KLRG1^+^ NK cell expression of CCR2. However, qPCR analysis revealed only very low-level expression of Ackr2 (Fig. 6A) that was at the threshold of detection. In addition, ligand binding assays (Fig. 6B) failed to demonstrate significant levels of Ackr2 activity on these cells above the background level detected on Ackr2^−/−^ KLRG1^+^ NK cells. To further investigate potential cell-autonomous effects of Ackr2, we used lentiviruses to overexpress Ackr2 in CCR2^+^ cells (Fig. 6C). Given the difficulties of transfecting/transducing NK cells and the fact that we have not previously identified cell-specific differences in Ackr2 function, we performed these experiments in CCR2^+^ J774.2 cells. Analysis of these cells by PCR (Fig. 6C) or flow cytometry (Fig. 6D) demonstrated no impact of Ackr2 on CCR2 levels. Together, these data suggest that Ackr2 is unlikely to exert a cell-autonomous effect in KLRG1^+^ NK cells.

### NK cells from Ackr2^−/−^ mice have enhanced tumor-homing efficiency

**FIGURE 7.**
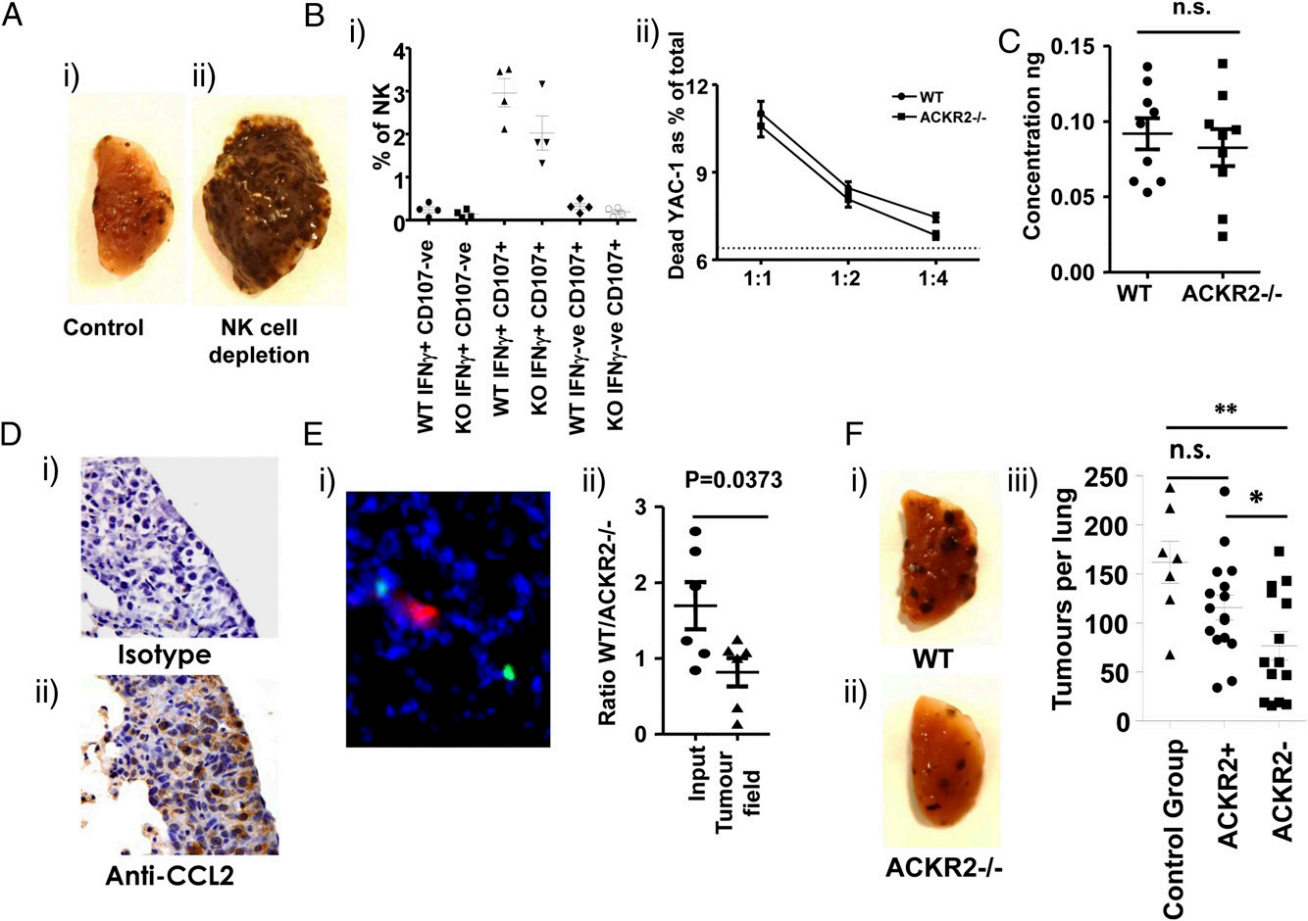
NK cells from Ackr2^−/−^ mice have enhanced tumor-homing efficiency. (**A**) Ab-based NK cell depletion (WT mice) enhances metastasis. (A**i**) Isotype, (A**ii**) NK cell–depleting Ab (10 mice per group). (**B**) WT and Ackr2^−/−^ KLRG1^+^ NK cells display equivalent target cell–killing ability measured by (B**i**) IFN-γ and CD107 expression (*n* = 4) and (B**ii**) YAC-1 target cell–killing (*n* = 4). (**C**) Resting WT and Ackr2^−/−^ lungs display equivalent CCL2 levels (ELISA of tissue homogenates) (*n* = 9). (**D**) Metastatic colonies display strong CCL2 expression. (D**i**) Isotype, (D**ii**) anti-CCL2 Ab. (**E**) Differentially labeled WT and Ackr2^−/−^ KLRG1^+^ NK cells were transferred to WT mice 72 h after labeled B16F10 administration and proximity of WT and Ackr2^−/−^ KLRG1^+^ NK cells to metastatic deposits were assessed. Original magnification ×5. (E**i**) Field (original magnification ×40) stained with DAPI (blue) and showing tumor cell (red) with neighboring Ackr2^−/−^ KLRG1^+^ NK cell (green). (E**ii**) WT/Ackr2^−/−^ cell ratio at input and fields incorporating tumor deposits. Reduced ratio indicates preferential Ackr2^−/−^ KLRG1^+^ NK cell presence in the indicated field (*n* = 6). One-way ANOVA, Tukey posttest. (**F**) WT mice injected with B16F10 cells, followed by WT or Ackr2^−/−^ KLRG1^+^ NK cells (isolated from spleens). Lung metastases were visualized (F**i** and F**ii**) and enumerated (F**iii**) at day 14 (*n* ≥ 14). The control group represents metastasis numbers in WT mice that have not been adoptively transferred with NK cells. All experiments were performed at least twice with similar results. *p = 0.05, unpaired *t* test.

As shown (Fig. 7A), and as previously reported (30), Ab-mediated NK cell depletion dramatically increased tumor development in WT mice, confirming a nonredundant role for NK cells in limiting metastasis in the B16F10 model. Further analysis of pulmonary metastasis development in WT and Ackr2^−/−^ mice treated with NK cell–depleting Abs revealed that with both genotypes, the lungs were full of tumor with extensive associated hemorrhage (Supplemental Fig. 4A). Quantification of the percentage of total lung area covered by tumor revealed no significant difference between WT and Ackr2^−/−^ lungs (Supplemental Fig. 4B). These data therefore support the nonredundant role for NK cells in restricting metastasis development in both genotypes. We next directly investigated the impact of enhanced CCR2 responsiveness of KLRG1^+^ NK cells from Ackr2^−/−^ mice on antitumor responses. KLRG1^+^ NK cells from Ackr2^−/−^ mice displayed cell-killing activity indistinguishable from that of WT cells using either the common NK cell target cell line YAC-1 (31) (Fig. 7B) or, and in agreement with previous reports (32), B16F10 cells themselves (Supplemental Fig. 4C). Thus, increased ability of individual KLRG1^+^ NK cells to mediate tumor killing cannot account for impaired metastasis development in Ackr2^−/−^ mice. In addition, there were no significant differences in CCL2 levels in resting WT and Ackr2^−/−^ lungs (Fig. 7C) that might contribute to enhanced basal pulmonary trafficking of NK cells from Ackr2^−/−^ mice. Interestingly, although B16F10 cells grown in vitro do not express CCL2, immunostaining revealed strong CCL2 expression by developing B16F10 metastatic deposits and surrounding stroma (Fig. 7D), indicating that the process of tumor formation induces CCL2 expression in these cells. These data further suggest that the strong expression of CCL2 by the tumor deposits might favor recruitment of CCR2 overexpressing KLRG1^+^ NK cells from Ackr2^−/−^ mice to developing metastases. Indeed, analysis of the relative proximity of dye-labeled, adoptively transferred, KLRG1^+^ NK cells from WT and Ackr2^−/−^ mice to tumor deposits (Fig. 7E) indicated that NK cells from Ackr2^−/−^ mice migrated closer to CCL2-expressing tumors than WT cells. These data suggest that enhanced NK cell recruitment directly to sites of developing metastases explains impaired development in Ackr2^−/−^ mice. Finally, to formally demonstrate enhanced antitumor activity of KLRG1^+^ NK cells from Ackr2^−/−^ mice, we adoptively transferred either KLRG1^+^ NK cells from WT or Ackr2^−/−^ mice to WT mice in receipt of B16F10 cells. As shown (Fig. 7Fi, 7Fii), fewer tumors developed in WT mice receiving the KLRG1^+^ NK cells from Ackr2^−/−^ compared with WT mice. Quantification of tumor numbers per lung revealed that KLRG1^+^ NK cells from Ackr2^−/−^ mice were significantly better at suppressing tumor development than WT counterparts (Fig. 7Fiii). These data are also interesting in that they suggest that the enhanced CCR2 expression and associated increased recruitment of NK cells from Ackr2^−/−^ mice to the metastatic sites (Fig. 7E) is not diminished upon transfer to WT mice and is thus presumably stable. Thus, in comparison with WT KLRG1^+^ NK cells, KLRG1^+^ NK cells from Ackr2^−/−^ mice increase tumor killing through enhanced CCR2 responsiveness and resulting recruitment in closer proximity to CCL2-expressing tumor deposits.

## Discussion

Chemokines and their receptors are fundamental players in metastasis (2, 3, 9). In addition, it is clear that tumoricidal cells, such as NK cells, will navigate to sites of tumor development using chemokines and their receptors, although, to our knowledge, this axis has not been rigorously studied. In this study, we demonstrate that Ackr2^−/−^ mice display enhanced protection against pulmonary metastasis development. We further demonstrate that this is associated with increased CCR2 expression by KLRG1^+^ NK cells from Ackr2^−/−^ mice and attendant hyperresponsiveness of these cells to tumor-expressed CCL2. This results in enhanced NK cell recruitment to the tumor site and an associated increase in tumor cell killing. Importantly, and as discussed above, we do not see an increase in the total number of KLRG1^+^ NK cells in early-stage metastatic Ackr2^−/−^ lungs; only increased CCR2 expression and associated enhanced proximity to the metastatic sites are seen. We therefore propose that CCR2 is likely to play a specific role in movement of KLRG1^+^ NK cells within the lung toward metastatic deposits and not in their initial recruitment from the circulation.

It is notable that monocytes and other CCR2-expressing cells, including KLRG1^−^ NK cells, do not display enhanced CCR2 expression in Ackr2^−/−^ mice, arguing for a role for Ackr2 in regulating CCR2 specifically in KLRG1^+^ NK cells. The absence of increased CCR2 expression in the other leukocyte populations also argues against general transcriptional upregulation of CCR2 in Ackr2^−/−^ cells. At this stage, we are unclear as to the precise mechanism behind the upregulation of CCR2 in Ackr2^−/−^ KLRG1^+^ NK cells. However, previous analyses identified CCR2 as marking a discrete subpopulation of murine NK cells (33), and although we do not detect other phenotypic differences between WT and Ackr2^−/−^ KLRG1^+^ NK cells, our current hypothesis is that the elevated CCR2 expression is an indication of a subtle variation in NK cell maturation in Ackr2^−/−^ mice. This suggestion is supported by recent data indicating elevated CCR2 expression in NK cells with an altered maturation profile (34). We note a recent publication confirming our metastasis observations that suggests that deletion of expression of Ackr2 in hematopoietic progenitor cells results in CCR2 upregulation in a variety of mature myeloid cells (35). However, Ackr2 expression is not reported in hematopoietic progenitor cells in either the ImmGen (ccbp2 gene designation in www.immgen.org) or Gene Expression Commons (Ackr2 gene designation in https://gexc.riken.jp) databases, and using sensitive PCR-based approaches, we have also been unable to detect Ackr2 in hematopoietic progenitor cells. We therefore do not believe that this is a plausible explanation for the phenotype reported and, as noted above, favor a model in which CCR2 elevation is explained by a subtle but specific alteration in NK cell maturation.

Our data provide an explanation for previous observations regarding the association of CCL2 and other chemokines with metastasis. For example, high serum CCL2 is associated with increased metastasis and poor overall survival in nasopharyngeal carcinoma (36). We suggest that this may relate to systemic downregulation of CCR2 on circulating tumoricidal leukocytes such as NK cells. Furthermore, deletion of CCL2 or CCL3 in mice is associated with decreased NK cell (and other leukocyte) attraction to, and inhibition of, metastatic lesions (37). These results are in keeping with the findings of the current study.

In contrast, some of our data conflict with previously published work. For example, our data show that unlike other models (9, 38, 39), CCR2-dependent macrophage accumulation at sites of metastatic cell extravasation is not essential for tumor development in the B16F10 model. Indeed, we routinely see metastasis developing in this model on a CCR2^−/−^ background. Nevertheless, Fig. 3B demonstrates a requirement for macrophages, suggesting alternative chemokine receptor involvement in prometastatic macrophage recruitment. Importantly, without this independence from CCR2-dependent macrophage involvement in metastatic cell extravasation, we would have been unable to determine the distinct roles for CCR2 in NK cell–mediated antitumor responses. Our data, therefore, highlight an additional level of complexity in the involvement of CCR2 (and potentially other inflammatory chemokine receptors) in regulating metastasis development.

Our study also highlights novel therapeutic options. Although, as shown in Fig. 6, Ackr2 does not appear to regulate NK cell CCR2 expression on a cell-autonomous basis, it is clearly involved in determining CCR2 expression through, for example, subtle alteration of NK cell maturation. If this altered maturation could be recapitulated by therapeutic systemic impairment of Ackr2 function, then this may lead to increased CCR2 expression by patient NK cells and enhance their antimetastatic potential. Furthermore, and given that CCL2 expression is a common feature of tumors (3), our data suggest that enhancing CCR2 expression by NK cells will increase their migration into tumor sites and enhance killing of nascent metastatic deposits.

In summary, we demonstrate profoundly impaired metastasis development in Ackr2^−/−^ mice and highlight enhanced NK cell responsiveness to CCR2 as the molecular basis for this. We believe that our study expands our knowledge of chemokine involvement in metastasis and has clear therapeutic potential.

## Supplementary Material

Data Supplement
