# Supplementary material for: The Atypical Chemokine Receptor Ackr2 Constrains NK Cell Migratory Activity and Promotes Metastasis
Source: J Immunol. 2018 Aug 29;201(8):2510–9. doi: 10.4049/jimmunol.1800131 (PMC6176105; doi:10.4049/jimmunol.1800131)
Supplement: Data Supplement [file JI_1800131.zip › JI_1800131_Supplemental_Figures_1.pdf]

Supplemental Information

The atypical chemokine receptor, Ackr2, constrains NK cell migratory activity and promotes metastasis. Hansell et al.

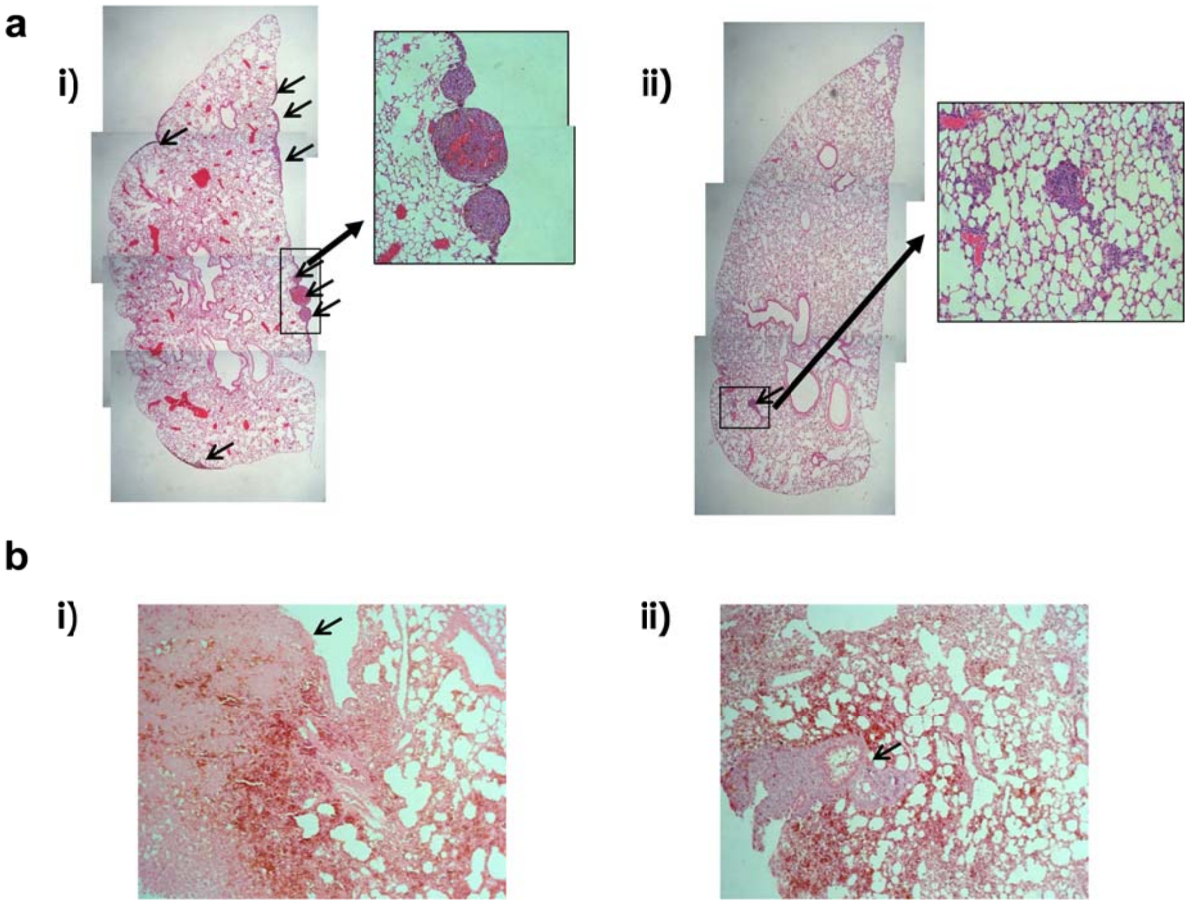

Figure S1. Low magnification images of lungs

These images are from mice on a) the B16F10 model and b) the Lewis Lung Carcinoma model. In both a and b, images in i) are from WT mice and ii) from Ackr2-/- mice. Tumour deposits are indicated by the arrows.

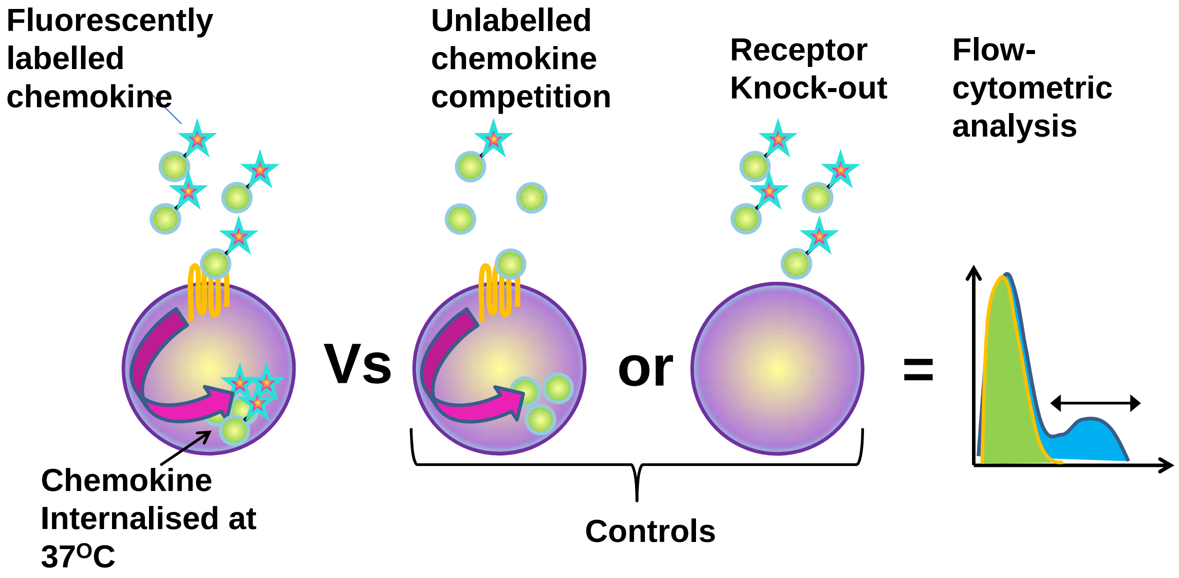

Figure S2. The Chemokine-uptake assay for assessment of receptor expression levels.

Isolated cells are cultured at 37^{0}C in the presence of fluorescently labelled chemokine. Cells with appropriate receptors internalise this chemokine and become fluorescently labelled. The level of fluorescence can be measured by flow cytometry. The specificity of the receptor can be determined, and non-specific mechanisms ruled out, through a comparison with control samples generated using either an excess of unlabelled competitor or derived from receptor knock-out mice. To detect CCR2 we typically use fluorescently labelled (Alexa-647) CCL2 with unlabelled CCL12 as competitor. To detect Ackr2 we use fluorescently labelled CCL22 with unlabelled CCL12 as competitor.

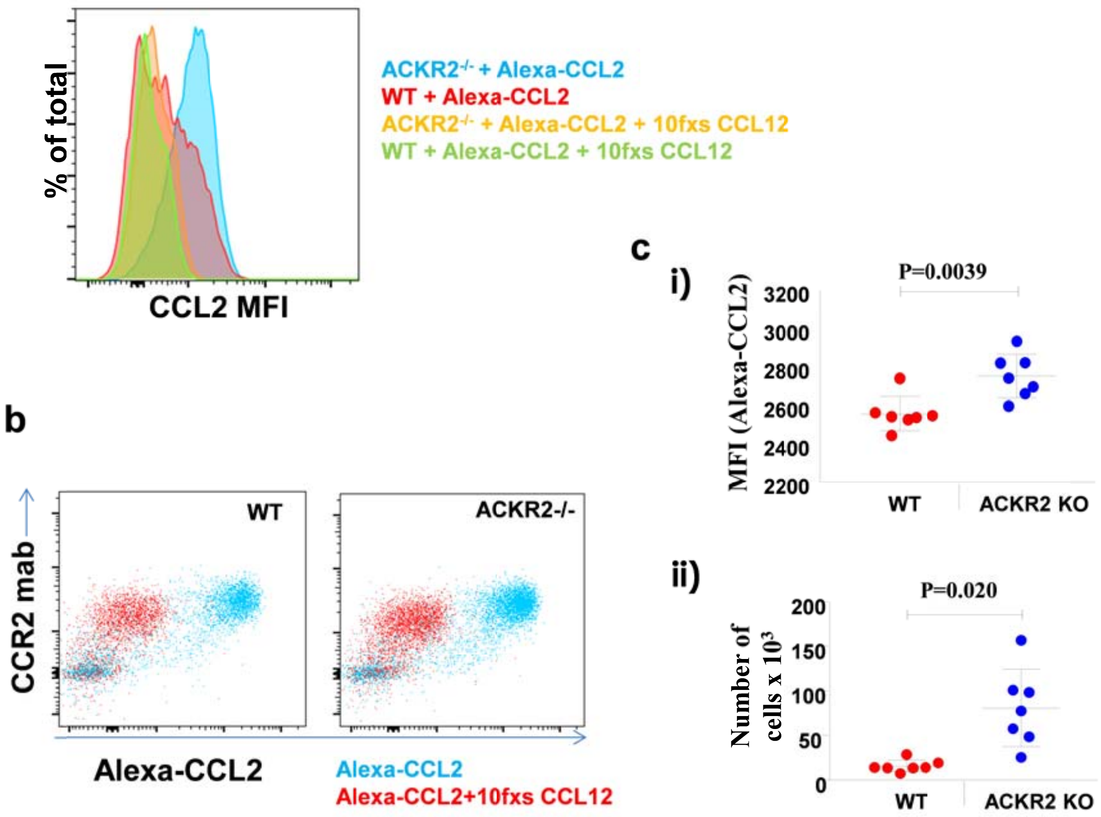

Figure S3: ACKR2-/- NK cells, but not monocytes, display increased levels of CCR2 expression.

a) CCR2 activity on WT and ACKR2-/- KLRG1+ NK cells was assessed by measuring the binding of Alexa-CCL2 in the presence or absence of CCL12 as a CCR2 specific competitor. This experiment was performed 2 times with similar results.

b) Flow cytometry data demonstrating identical binding of Alexa-CCL2 (+/- CCL12 competition) by WT and ACKR2-/- monocytes and full overlap with anti-CCR2 antibody binding. This experiment was performed 2 times with similar results. The anti-CCR2 antibody was not used with NK cells in a) as it does not detect cell surface CCR2 expression in these cells. In addition the antiCCR2 antibody does not compete with ligand for receptor binding(14).

C) Analysis of CCR2 expression by (i) and numbers of (ii) KLRG1+ NK cells in the lungs of WT and Ackr2-/- mice at the termination of the B16F10 metastasis analysis (day 14).

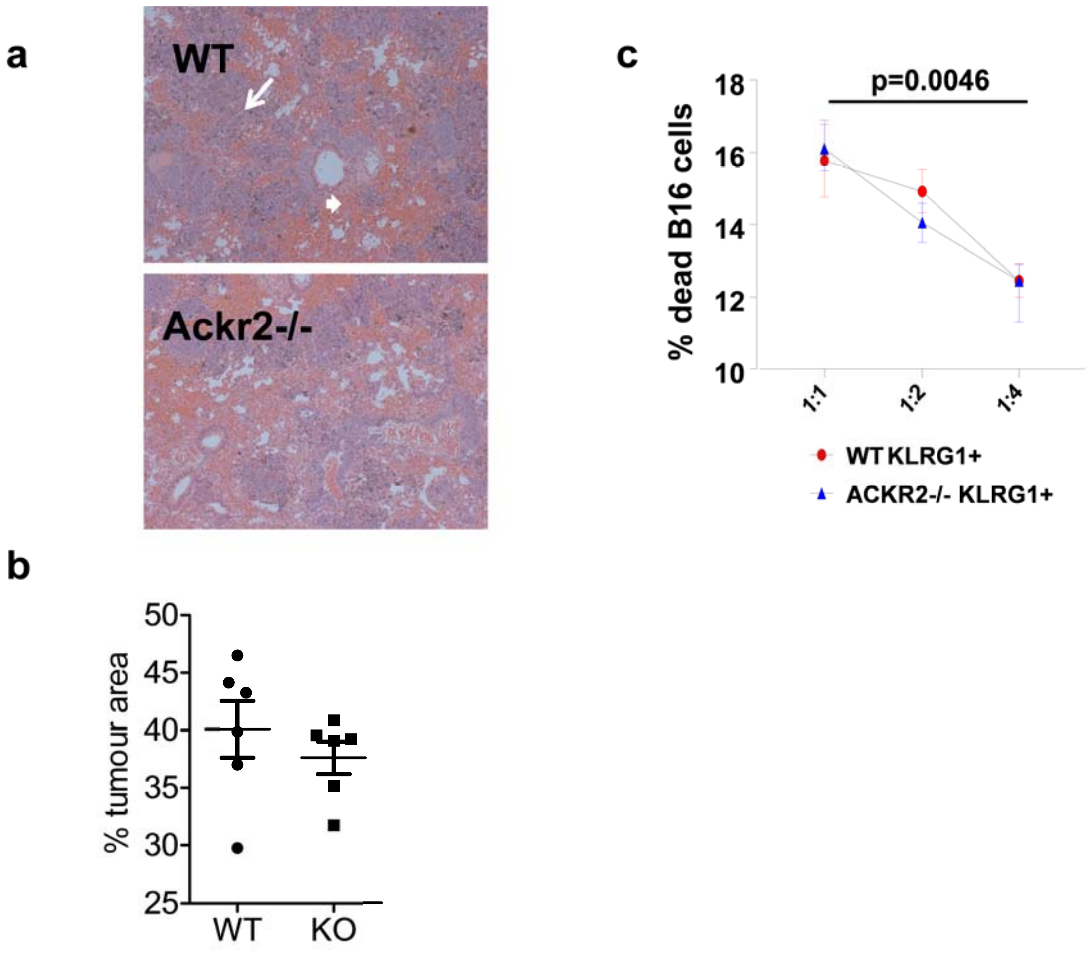

Figure S4: NK cell depleting antibody treatment massively increases the tumor burden in WT and Ackr2-/- mice receiving B16-F10 cells.

a) Histological images of lung sections from WT and Ackr2-/- mice showing extensive tumor areas (arrow) and associated haemorrhage (arrow head).

b) Quantitative assessment of the cross-sectional area of the lung taken up by tumor in WT and Ackr2-/- mice treated with NK cell depleting antibodies. These data are not significantly different.

C) Killing of B16F10 cells by WT and Ackr2-/- KLRG1+ NK cells.
